# Architectural planning robot driven by unsupervised learning for space optimization

**DOI:** 10.3389/fnbot.2024.1517960

**Published:** 2025-01-03

**Authors:** Zhe Zhang, Yuchun Zheng

**Affiliations:** ^1^College of Resources and Environment, Fujian Agriculture and Forestry University, Fuzhou, Fujian, China; ^2^Department of Architectural Engineering, Jinhua Polytecnich, Jinhua, Zhejiang, China

**Keywords:** space optimization, architectural planning, unsupervised learning, spatial attention, clustering

## Abstract

**Introduction:**

Space optimization in architectural planning is a crucial task for maximizing functionality and improving user experience in built environments. Traditional approaches often rely on manual planning or supervised learning techniques, which can be limited by the availability of labeled data and may not adapt well to complex spatial requirements.

**Methods:**

To address these limitations, this paper presents a novel architectural planning robot driven by unsupervised learning for automatic space optimization. The proposed framework integrates spatial attention, clustering, and state refinement mechanisms to autonomously learn and optimize spatial configurations without the need for labeled training data. The spatial attention mechanism focuses the model on key areas within the architectural space, clustering identifies functional zones, and state refinement iteratively improves the spatial layout by adjusting based on learned patterns. Experiments conducted on multiple 3D datasets demonstrate the effectiveness of the proposed approach in achieving optimized space layouts with reduced computational requirements.

**Results and discussion:**

The results show significant improvements in layout efficiency and processing time compared to traditional methods, indicating the potential for real-world applications in automated architectural planning and dynamic space management. This work contributes to the field by providing a scalable solution for architectural space optimization that adapts to diverse spatial requirements through unsupervised learning.

## 1 Introduction

Optimizing architectural spaces and 3D reconstruction are pivotal challenges in modern urban planning and building design (Wang et al., 2022). These tasks play a fundamental role in ensuring the efficient use of spatial resources, improving functionality, and minimizing costs, while also enabling dynamic reconfiguration to adapt to evolving demands (Wu et al., 2022). Beyond their direct benefits to building design, advancements in these domains can significantly enhance energy efficiency and sustainability, making them critical for addressing global environmental goals. Additionally, the integration of 3D reconstruction facilitates accurate digital modeling of architectural structures, supporting detailed analysis, renovation, and automation (Liu et al., 2023). Despite their significance, the current approaches to architectural space optimization face several key challenges:The ability to handle diverse and non-standard spatial configurations remains limited. Existing methods often lack adaptability to dynamic requirements or real-time adjustments. High computational costs and the lack of transparency in complex models hinder practical applications (Wang et al., 2021).

Traditional solutions, such as rule-based and symbolic AI methods, have long dominated the field (Amice et al., 2022). These techniques codify expert knowledge into structured systems that optimize spatial layouts based on predefined criteria. While these approaches excel in ensuring compliance with architectural standards, they suffer from rigidity and a lack of scalability (Li et al., 2022). As spatial configurations grow more complex, symbolic methods require extensive manual adjustments, reducing their practicality for large-scale or rapidly evolving scenarios (Kästner et al., 2023). Machine learning and data-driven approaches introduced a new paradigm by leveraging statistical models to identify patterns in spatial data (Liu et al., 2020). Techniques such as clustering and regression improved the flexibility of layout optimization, while machine learning models like decision trees and support vector machines offered predictive capabilities based on historical data (Xie et al., 2020). However, these methods still heavily rely on high-quality labeled datasets, which are often scarce or expensive to obtain. Consequently, their generalization to unseen or unconventional layouts remains limited (Pan et al., 2022). To address these gaps, deep learning approaches such as convolutional neural networks (CNNs; Zheng et al., 2022) and generative adversarial networks (GANs; Liu et al., 2022) have been adopted for space optimization and 3D reconstruction. These models demonstrate significant potential in capturing complex spatial features and adapting to various tasks through pre-training (Beach et al., 2023). Despite these advancements, deep learning methods introduce new challenges, including high computational demands and limited interpretability. Their black-box nature restricts their application in domains requiring adherence to rigorous standards or transparency (Vieira et al., 2022).

In order to enhance the cross-value between this article and LLM technology, we have added several latest research documents and compared and combined them with the method of this article. Chronis et al. (2024) proposed a robot task execution framework based on LLM and scene graphs, demonstrating the powerful capabilities of LLM in scene understanding and dynamic task planning. Chugh et al. (2024) proposed dynamic path planning for autonomous robots based on dynamic graphs and breadth-first search, which provides an important reference for applying path planning optimization in dynamic architectural environments. In addition, Wang et al. (2023) demonstrated a method of generating cues through a large language model to guide robot gait task planning, demonstrating the advantages of LLM in complex task adaptability and semantic understanding.

Recognizing the limitations of these methods, this paper proposes a novel framework that leverages unsupervised learning, modular design principles, and adaptive spatial attention to address the key issues in architectural space optimization and 3D reconstruction. Unsupervised learning eliminates the need for labeled data, enabling the model to generalize across diverse scenarios. The modular design facilitates the integration of multiple optimization techniques, ensuring scalability and adaptability. Finally, the adaptive spatial attention mechanism dynamically focuses computational resources on the most relevant spatial features, reducing costs while improving accuracy.

The contributions of this work include:

- A dynamic system for prioritizing critical architectural regions, enhancing space utilization efficiency.
- A design that supports adaptation to various contexts, ensuring high generalizability.
- Experimental results highlight superior performance in layout optimization and 3D reconstruction compared to state-of-the-art methods, along with significant reductions in computational costs.

## 2 Related work

### 2.1 Traditional space optimization techniques

Traditional approaches to space optimization in architectural planning often rely on manual methods or heuristic algorithms. Manual methods involve human experts who utilize architectural principles and spatial requirements to create optimal layouts (Zhang et al., 2021). While effective for simple projects, these methods become increasingly impractical as the complexity of architectural requirements grows. Heuristic algorithms, such as simulated annealing, genetic algorithms, and particle swarm optimization, have been used to automate parts of the design process (Atzori et al., 2016). These algorithms aim to find near-optimal solutions by iteratively refining the space configuration based on predefined objective functions. Although they can improve layout efficiency, heuristic methods still require careful tuning of parameters and a good understanding of the underlying problem, which can limit their adaptability to diverse and changing spatial requirements (Marcucci et al., 2022). Another drawback of traditional space optimization techniques is their limited ability to handle real-time or dynamic space modifications (Jin et al., 2024b). In scenarios where space usage evolves frequently—such as co-working spaces, hospitals, or smart homes—manual and heuristic approaches may struggle to adapt quickly enough to meet new requirements. Furthermore, these methods typically do not account for the spatial relationships and functional interactions between different regions, which can lead to suboptimal configurations in complex environments. The emergence of artificial intelligence (AI) techniques has introduced more sophisticated methods for addressing these limitations, laying the groundwork for the integration of machine learning and automated planning in architectural space optimization (Cauligi et al., 2020).

### 2.2 Supervised learning for space planning

In recent years, machine learning techniques, particularly supervised learning, have been applied to architectural space optimization. Supervised learning methods leverage large datasets of labeled examples to train models that can predict optimal space configurations based on input features such as building layouts, user preferences, and functional requirements. Techniques such as convolutional neural networks (CNNs) and recurrent neural networks (RNNs) have been used to process spatial data and generate layout proposals. These models learn spatial patterns from the training data and can be fine-tuned to meet specific objectives, such as maximizing natural light, improving accessibility, or minimizing energy consumption (Hu et al., 2023). However, supervised learning approaches have several limitations in the context of space optimization. The requirement for large amounts of labeled data can be a significant drawback, as gathering and annotating spatial datasets is time-consuming and expensive (Li et al., 2024). The models trained using supervised learning may not generalize well to new or unseen architectural scenarios, especially when the training data does not cover the full range of possible spatial configurations. As a result, supervised models may perform poorly in environments with highly variable or unconventional layout requirements. Furthermore, the reliance on labeled data makes it challenging for supervised methods to adapt in real-time to evolving spatial needs, as new training data must be collected and labeled for the model to remain effective. These limitations have led researchers to explore unsupervised and semi-supervised learning techniques for more flexible and adaptive space optimization solutions (Jin et al., 2024a).

### 2.3 Unsupervised learning and self-organizing systems

Unsupervised learning approaches and self-organizing systems have gained attention in the field of architectural space optimization as a means to overcome the limitations associated with supervised learning. Unsupervised learning techniques do not require labeled data, making them suitable for scenarios where data collection and annotation are difficult. Methods such as clustering, dimensionality reduction, and generative models (Zhang et al., 2024) have been employed to learn underlying patterns in spatial data and generate optimal configurations. Clustering techniques, for instance, can be used to identify functional zones within a space based on user behavior or environmental factors, while dimensionality reduction can help visualize complex spatial relationships in a lower-dimensional space (Chang et al., 2023). Self-organizing systems, inspired by natural processes such as the growth of biological tissues or the behavior of ant colonies, offer another approach to space optimization. These systems utilize local rules or interactions between agents to achieve global spatial organization without centralized control. For example, agent-based modeling can simulate the behavior of occupants in a space to optimize room configurations based on predicted usage patterns. Similarly, self-organizing maps (SOMs) have been used to arrange spaces based on similarity criteria, enabling adaptive and emergent design solutions (Hewawasam et al., 2022). Despite their potential, unsupervised learning and self-organizing systems still face challenges in architectural applications. The quality of the generated solutions heavily depends on the choice of algorithm and parameters, which may require domain-specific knowledge. The interpretability of results can be a concern, as the models do not explicitly learn to optimize for predefined objectives like supervised methods (Spahn et al., 2021). Nonetheless, these approaches offer significant advantages in terms of flexibility and adaptability, making them promising candidates for real-time and dynamic space optimization tasks (Jin et al., 2023).

## 3 Methodology

### 3.1 Overview

**Figure 1:**
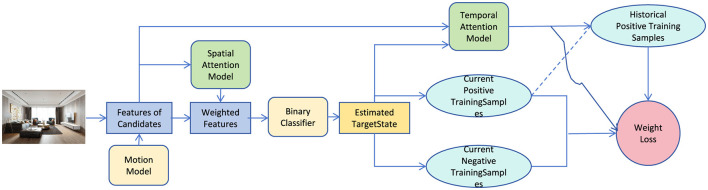
The proposed overall framework. Through the spatial attention model, binary classifier and temporal attention model, the current positive and negative training samples are generated, and the weights are optimized through historical samples.

The proposed architectural planning robot framework leverages unsupervised learning for optimizing indoor spatial layouts, focusing on dynamic space utilization and adaptation to evolving requirements. The system integrates a Convolutional Neural Network (CNN) for feature extraction from visual inputs, such as floor plans, to capture spatial features and functional zones. Using neural robotics technology, the robot autonomously adjusts the architectural design in real-time based on user interactions and environmental changes, allowing for continuous space optimization. The unsupervised learning approach eliminates the need for labeled training data, making it suitable for a variety of planning scenarios where spatial requirements frequently change. The framework incorporates multiple modules: feature extraction through CNNs, spatial reasoning based on dynamic attention mechanisms, and a feedback-driven design refinement process. In sub-section 3.2, we elaborate on the feature extraction mechanism, while sub-section 3.3 discusses the design refinement and optimization strategies. Sub-section 3.6 covers the incorporation of real-time user and environmental feedback for adaptive planning (as shown in Figure 1).

This paper introduces unsupervised learning technology to significantly reduce the need for large-scale annotated data. Traditional deep learning methods rely on high-quality annotated data to train models, which is often costly and time-consuming in complex scenarios. The method in this article automatically identifies and optimizes the spatial layout by learning latent spatial patterns in unlabeled data, which not only improves the adaptability of the model, but also significantly reduces data requirements. In addition, the modular design allows the system to flexibly adjust the computing resource allocation of sub-modules, avoiding the waste of computing that treats the entire scene equally, thus optimizing computing efficiency. The adaptive spatial attention mechanism further reduces redundant calculations by focusing on key areas to ensure efficient use of resources. In response to the problem of insufficient model interpretability, the modularization and attention mechanism design of this article provide higher transparency. The modular design clarifies the function of each sub-module in the overall optimization of the model. For example, the spatial attention mechanism points to key areas that need priority optimization, making the logic of the model output more interpretable. In addition, the experimental part further demonstrates the specific functions of these modules through visual attention distribution and performance analysis, providing intuitive support for understanding model behavior.

### 3.2 Preliminaries

The problem of architectural space optimization can be formulated as a dynamic optimization task, where the goal is to maximize the utility of an indoor space by adjusting its layout in response to varying constraints and objectives. Let the architectural space be represented by a set of spatial regions *S* = {*s*_1_, *s*_2_, …, *s*_*n*_}, where each region *s*_*i*_ is characterized by its geometric properties (dimensions, shape), functional requirements, and adjacency relationships with other regions. The layout configuration at a given time can be defined by a set of variables *X* = {*x*_1_, *x*_2_, …, *x*_*n*_}, where each *x*_*i*_ represents the position and orientation of region *s*_*i*_.

The optimization task aims to find the optimal configuration X^*^ that maximizes a utility function *U*(*X*) subject to a set of constraints. The utility function is defined as:


(1) $$\begin{array}{l} U\left(X\right)=\overset{n}{\underset{i=1}{\sum }}w_{i}\cdot f_{i}\left(x_{i}\right), \end{array}$$

where *f*_*i*_(*x*_*i*_) denotes the utility of region *s*_*i*_ based on its configuration *x*_*i*_, and *w*_*i*_ represents a weighting factor reflecting the importance of each region. The utility function can incorporate various criteria such as accessibility, natural lighting, privacy, and functionality, which are crucial for achieving an optimal layout.

The constraints in this optimization problem can be divided into geometric constraints and functional constraints. Geometric constraints ensure that regions do not overlap and maintain appropriate distances, while functional constraints ensure compliance with spatial requirements, such as minimum room size or specific adjacency relationships. These constraints can be mathematically expressed as:


(2) $$\begin{array}{l} g_{j}\left(X\right)\leq 0,\;j=1,\ldots ,m, \end{array}$$

where *g*_*j*_(*X*) represents a constraint function that the layout must satisfy.

To solve this optimization problem in a dynamic environment where spatial requirements may change, the framework employs a multi-objective optimization approach. The objectives can be adjusted in real-time based on user feedback and environmental conditions. The optimization is thus represented as:


(3) $$\begin{array}{l} \underset{X}{\max}U\left(X\right),\;\text{subject to }g_{j}\left(X\right)\leq 0,j=1,\ldots ,m. \end{array}$$

To model the changing spatial requirements, we introduce a time-dependent component *X*(*t*) that allows the configuration to evolve over time. The time evolution of the layout can be represented using a differential equation:


(4) $$\begin{array}{l} \frac{dX\left(t\right)}{dt}=F\left(X\left(t\right),t\right), \end{array}$$

where *F*(*X*(*t*), *t*) denotes the update function that adjusts the layout based on the current state and external influences.

The unsupervised learning component is employed to learn spatial patterns from unlabeled data. Given a set of spatial configurations {*X*_1_, *X*_2_, …, *X*_*T*_} over time, the learning objective is to capture the underlying distribution *P*(*X*) that characterizes optimal spatial arrangements. This can be achieved using clustering techniques or generative models, such as autoencoders, which learn to represent the data in a lower-dimensional space while preserving the essential spatial relationships.

The framework also incorporates a feedback mechanism that continuously refines the learned spatial patterns based on user interactions and real-time sensor data. The feedback process can be formalized as:


(5) $$\begin{array}{l} X_{new}=X_{prev}+\alpha \cdot \nabla U\left(X_{prev}\right), \end{array}$$

where *X*_*prev*_ is the previous configuration, α is a learning rate, and ∇*U*(*X*_*prev*_) is the gradient of the utility function with respect to the configuration. This feedback loop enables the system to adapt to changes in spatial requirements and improve the layout iteratively.

### 3.3 Feature extraction with spatial attention

#### 3.3.1 Adaptive visibility mapping

To enhance the robustness of spatial feature extraction, we introduce an Adaptive Visibility Mapping mechanism. This method aims to address occlusion and distortion issues that arise in candidate state representations during real-time architectural space optimization. By leveraging a multi-stage convolutional architecture, the method ensures precise visibility estimation and adaptability to dynamic spatial configurations.

The visibility map $\text{V}\left({\text{y}}_{l}^{m}\right)\in {\mathbb{R}}^{A\times B}$ for a candidate state ${\text{y}}_{l}^{m}$ is generated through a hierarchical process designed to capture fine-grained spatial features. The visibility estimation is computed as:


(6) $$\begin{array}{l} \text{V}\left({\text{y}}_{l}^{m}\right)=g_{\text{vis}}\left({\text{Φ}}_{\text{roi}}\left({\text{y}}_{l}^{m}\right);{\text{W}}_{\text{vis}}\right), \end{array}$$

where *g*_vis_ is a visibility function implemented using a cascade of convolutional layers with ReLU activation and batch normalization. The parameter set **W**_vis_ includes the weights and biases of these layers. This setup allows $\text{V}\left({\text{y}}_{l}^{m}\right)$ to emphasize regions with high visibility while suppressing noisy or occluded areas.

To account for the inherent spatial correlations in architectural layouts, we include a spatial regularization term. This term ensures that abrupt variations in visibility between neighboring pixels are minimized, leading to smoother and more coherent visibility maps:


(7) $$\begin{array}{l} L_{\text{reg}}\left(\text{V}\right)=\lambda \underset{i,j}{\sum }\left({\left({\text{V}}_{i+1,j}-{\text{V}}_{i,j}\right)}^{2}+{\left({\text{V}}_{i,j+1}-{\text{V}}_{i,j}\right)}^{2}\right), \end{array}$$

where λ is a regularization coefficient that balances the trade-off between smoothness and feature fidelity. The summation iterates over all spatial locations (*i, j*) in the visibility map **V**.

Furthermore, the adaptive aspect of the visibility mapping is achieved through a multi-resolution refinement strategy. Initial visibility maps are generated at a coarse resolution and iteratively refined to higher resolutions using a learned refinement network:


(8) $$\begin{array}{l} {\text{V}}_{k+1}\left({\text{y}}_{l}^{m}\right)={\text{V}}_{k}\left({\text{y}}_{l}^{m}\right)+h_{\text{ref}}\left({\text{V}}_{k}\left({\text{y}}_{l}^{m}\right);{\text{W}}_{\text{ref}}\right), \end{array}$$

where **V**_*k*_ denotes the visibility map at resolution level *k*, and *h*_ref_ is a refinement function parameterized by **W**_ref_. This iterative process ensures that details missed in the initial estimation are progressively captured.

To further enhance the interpretability of the visibility maps, a confidence score $c\left({\text{y}}_{l}^{m}\right)$ is computed for each candidate state. The score is derived by aggregating the visibility values within the region of interest:


(9) $$\begin{array}{l} c\left({\text{y}}_{l}^{m}\right)=\frac{1}{A\times B}\overset{A}{\underset{i=1}{\sum }}\overset{B}{\underset{j=1}{\sum }}{\text{V}}_{i,j}\left({\text{y}}_{l}^{m}\right). \end{array}$$

This confidence score is used as an auxiliary signal in downstream tasks such as classification and spatial reasoning, enabling the model to prioritize candidate states with higher visibility.

### 3.4 Multi-Scale Spatial Attention integration

**Figure 2:**
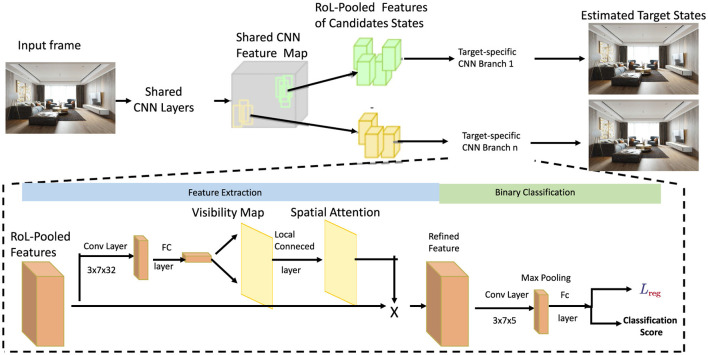
Framework diagram of feature extraction with spatial attention. The candidate state features are extracted from the shared CNN layer, the target state is estimated by calculating the target-specific CNN branch, and visibility and spatial attention are processed by the feature extraction module, finally achieving binary classification and loss optimization.

Building upon the visibility maps, we introduce a Multi-Scale Spatial Attention (MSSA) mechanism designed to capture and emphasize significant spatial regions across multiple resolutions. This approach enhances the feature representation by integrating information from various spatial scales, ensuring that both macro and micro architectural elements are effectively captured (as shown in Figure 2).

The attention map $\text{A}\left({\text{y}}_{l}^{m}\right)$ for a candidate state ${\text{y}}_{l}^{m}$ is generated by aggregating multi-scale feature maps:


(10) $$\begin{array}{l} \text{A}\left({\text{y}}_{l}^{m}\right)=\overset{S}{\underset{s=1}{\sum }}w_{s}\cdot h_{\text{att},s}\left({\text{V}}_{s}\left({\text{y}}_{l}^{m}\right);{\text{W}}_{\text{att},s}\right), \end{array}$$

where *w*_*s*_ are learnable weights assigned to each scale *s*, *S* denotes the number of scales, and *h*_att, *s*_ is a scale-specific function parameterized by **W**_att, *s*_. The use of learnable weights *w*_*s*_ allows the model to dynamically prioritize scales that are most relevant to the current architectural layout.

To generate the scale-specific feature map ${\text{V}}_{s}\left({\text{y}}_{l}^{m}\right)$, the input feature map is processed through a sequence of down-sampling and up-sampling operations. For a given scale *s*, the feature map is obtained as:


(11) $$\begin{array}{l} {\text{V}}_{s}\left({\text{y}}_{l}^{m}\right)=f_{\text{down},s}\left({\text{Φ}}_{\text{roi}}\left({\text{y}}_{l}^{m}\right);{\text{W}}_{\text{down},s}\right), \end{array}$$

where *f*_down, *s*_ is a convolutional down-sampling operation parameterized by **W**_down, *s*_. The feature map is then up-sampled back to the original resolution using:


(12) $$\begin{array}{l} {\text{V}}_{s}^{\text{up}}\left({\text{y}}_{l}^{m}\right)=f_{\text{up},s}\left({\text{V}}_{s}\left({\text{y}}_{l}^{m}\right);{\text{W}}_{\text{up},s}\right), \end{array}$$

where *f*_up, *s*_ is an up-sampling operation parameterized by **W**_up, *s*_. These operations ensure that spatial information at different scales is uniformly represented in the attention map computation.

To combine the scale-specific maps, a normalization step is applied to maintain numerical stability:


(13) $$\begin{array}{l} \hat{\text{A}}\left({\text{y}}_{l}^{m}\right)=\frac{\text{A}\left({\text{y}}_{l}^{m}\right)}{\sum_{i,j}{\text{A}}_{i,j}\left({\text{y}}_{l}^{m}\right)+\epsilon }, \end{array}$$

where ϵ is a small constant to prevent division by zero. The normalized attention map $\hat{\text{A}}\left({\text{y}}_{l}^{m}\right)$ ensures that the attention weights are bounded and interpretable.

To enhance feature representation further, the computed attention map is applied to the original feature map using an element-wise multiplication:


(14) $$\begin{array}{l} {\text{Φ}}_{\text{att}}\left({\text{y}}_{l}^{m}\right)={\text{Φ}}_{\text{roi}}\left({\text{y}}_{l}^{m}\right)⊙\hat{\text{A}}\left({\text{y}}_{l}^{m}\right), \end{array}$$

where ⊙ denotes the element-wise Hadamard product. This operation amplifies the features in regions of high attention while suppressing less relevant regions, improving the quality of extracted features.

To adapt dynamically to varying architectural layouts, a feedback mechanism is integrated. The feedback adjusts the attention weights *w*_*s*_ based on the classification error of the downstream task:


(15) $$\begin{array}{l} w_{s}^{\text{new}}=w_{s}^{\text{current}}-\eta \cdot \frac{\partial L_{\text{cls}}}{\partial w_{s}}, \end{array}$$

where η is the learning rate, and $L_{\text{cls}}$ is the classification loss. This adjustment ensures that the attention mechanism aligns with the broader objectives of the spatial optimization framework.

The spatial attention module plays a core role in the overall framework, and its function is reflected by its close connection with other modules. First, the module receives input features extracted by the "Features of Candidates Module," which combines the dynamic information generated by the motion model to represent the feature expressions of different regions or targets in the scene. Through the spatial attention mechanism, the module weights the input features, generates weighted features that reflect the focus of the model, and passes them to the binary classifier to predict the target state. At the same time, the weighted features are also combined with the Temporal Attention Model to use information from the time dimension to improve the ability to understand dynamic scenes. In addition, the classification results (Estimated Target State) are used to update the current positive and negative training sample sets (Current Positive/Negative Training Samples), which indirectly affects the adjustment and optimization of the attention module. The historical positive sample set (Historical Positive Training Samples) and the weight loss function (Weight Loss) further enhance the model's ability to focus on important features. It can be seen that the spatial attention module forms a collaborative optimization mechanism with the classifier and the temporal attention model by dynamically screening and weighting key features, thereby achieving more efficient classification and state prediction.

### 3.5 Dynamic Refinement and Feedback Mechanism

To adaptively refine attention maps during real-time optimization, we propose a Dynamic Refinement and Feedback Mechanism. This mechanism continuously updates spatial attention weights based on error signals derived from downstream classification tasks, ensuring that the attention mechanism aligns with the evolving architectural requirements and model objectives.

The attention refinement process starts by computing the updated attention map ${\text{A}}_{\text{new}}\left({\text{y}}_{l}^{m}\right)$ for a candidate state ${\text{y}}_{l}^{m}$ as:


(16) $$\begin{array}{l} {\text{A}}_{\text{new}}\left({\text{y}}_{l}^{m}\right)=\text{A}\left({\text{y}}_{l}^{m}\right)+\gamma \cdot \nabla_{\text{A}}L_{\text{cls}}, \end{array}$$

where $L_{\text{cls}}$ represents the classification loss, γ is the feedback learning rate, and $\nabla_{\text{A}}L_{\text{cls}}$ is the gradient of the loss with respect to the attention map. This gradient provides a direct signal for refining the spatial focus based on the classification task's performance.

To stabilize the feedback process and prevent over-correction, a momentum term β is introduced, resulting in a smoothed update:


(17) $$\begin{array}{l} \text{Δ}\text{A}\left({\text{y}}_{l}^{m}\right)=\beta \cdot \text{Δ}{\text{A}}_{\text{prev}}+\left(1-\beta \right)\cdot \gamma \cdot \nabla_{\text{A}}L_{\text{cls}}, \end{array}$$

(18) $$\begin{array}{l} {\text{A}}_{\text{new}}\left({\text{y}}_{l}^{m}\right)=\text{A}\left({\text{y}}_{l}^{m}\right)+\text{Δ}\text{A}\left({\text{y}}_{l}^{m}\right), \end{array}$$

where Δ**A**_prev_ is the update from the previous iteration. The momentum term ensures smoother transitions in the attention weights, avoiding abrupt changes that could destabilize the learning process.

Additionally, the updated attention map is normalized to maintain interpretability and numerical stability:


(19) $$\begin{array}{l} {\hat{\text{A}}}_{\text{new}}\left({\text{y}}_{l}^{m}\right)=\frac{{\text{A}}_{\text{new}}\left({\text{y}}_{l}^{m}\right)}{\sum_{i,j}{\text{A}}_{i,j}^{\text{new}}\left({\text{y}}_{l}^{m}\right)+\epsilon }, \end{array}$$

where ϵ is a small constant to prevent division by zero. This normalization ensures that the attention values remain bounded within a meaningful range.

To enhance the model's adaptability to varying spatial configurations, we incorporate a confidence-weighted feedback mechanism. Each candidate state is assigned a confidence score $c\left({\text{y}}_{l}^{m}\right)$, computed as:


(20) $$\begin{array}{l} c\left({\text{y}}_{l}^{m}\right)=\sigma \left(f_{\text{conf}}\left({\text{Φ}}_{\text{roi}}\left({\text{y}}_{l}^{m}\right);{\text{W}}_{\text{conf}}\right)\right), \end{array}$$

where *f*_conf_ is a function parameterized by **W**_conf_, and σ is the sigmoid function. The confidence score modulates the impact of the feedback on the attention map:


(21) $$\begin{array}{l} {\text{A}}_{\text{weighted}}\left({\text{y}}_{l}^{m}\right)=c\left({\text{y}}_{l}^{m}\right)\cdot {\hat{\text{A}}}_{\text{new}}\left({\text{y}}_{l}^{m}\right). \end{array}$$

The refined attention map is then used to compute the attention-weighted feature map:


(22) $$\begin{array}{l} {\text{Φ}}_{\text{att}}\left({\text{y}}_{l}^{m}\right)={\text{Φ}}_{\text{roi}}\left({\text{y}}_{l}^{m}\right)⊙{\text{A}}_{\text{weighted}}\left({\text{y}}_{l}^{m}\right), \end{array}$$

where ⊙ denotes the Hadamard product. This operation ensures that the extracted features emphasize the most relevant spatial regions identified through the feedback mechanism.

A temporal smoothing strategy is applied to the attention maps to incorporate historical information and reduce noise. The smoothed attention map is computed as:


(23) $$\begin{array}{l} \tilde{\text{A}}\left({\text{y}}_{l}^{m}\right)=\alpha \cdot {\text{A}}_{\text{weighted}}\left({\text{y}}_{l}^{m}\right)+\left(1-\alpha \right)\cdot {\tilde{\text{A}}}_{\text{prev}}\left({\text{y}}_{l}^{m}\right), \end{array}$$

where α is the smoothing factor, and ${\tilde{\text{A}}}_{\text{prev}}$ is the attention map from the previous time step. This temporal integration enhances robustness against short-term variations and improves consistency in feature extraction.

### 3.6 Innovative approaches in architectural planning robotics

#### 3.6.1 Unsupervised learning for spatial feature extraction

The proposed framework utilizes an unsupervised learning approach to extract and optimize spatial features, eliminating the need for labeled data. By leveraging Convolutional Neural Networks (CNNs) in conjunction with spatial attention mechanisms, the model dynamically identifies critical architectural features, such as boundaries, utilities, and structural elements, from diverse data sources, including 3D scans and blueprints. The CNN generates feature maps, Φ(I), which capture multi-scale spatial hierarchies crucial for understanding complex layouts:


(24) $$\begin{array}{l} \text{Φ}\left(I\right)=\text{CNN}\left(I\right), \end{array}$$

where I represents the input data. The spatial attention mechanism then prioritizes significant regions of these feature maps through dynamically learned weights:


(25) $$\begin{array}{l} \text{Ψ}=f_{\text{att}}\left(\text{Φ}\left(I\right)\right), \end{array}$$

and the refined feature map is computed as:


(26) $$\begin{array}{l} {\text{Φ}}_{\text{att}}=\text{Φ}\left(I\right)⊙\text{Ψ}, \end{array}$$

where ⊙ denotes element-wise multiplication. The framework incorporates feedback-driven adjustments to refine Ψ, allowing it to adapt to changing spatial configurations:


(27) $$\begin{array}{l} {\text{Ψ}}_{\text{new}}={\text{Ψ}}_{\text{current}}+\eta \cdot \nabla_{\text{Ψ}}U\left({\text{Φ}}_{\text{att}}\right), \end{array}$$

where ∇_Ψ_*U*(Φ_att_) represents the gradient of the utility function, ensuring dynamic updates. This unsupervised approach provides flexibility and scalability, making it ideal for architectural planning in evolving environments.

#### 3.6.2 Adaptive layout adjustment through clustering

To optimize the spatial layout, the framework employs a clustering-based strategy to identify functional zones within the extracted spatial features. This process segments the spatially-attentive feature map Φ_att_ into *k* clusters, {*C*_1_, *C*_2_, …, *C*_*k*_}, corresponding to distinct functional areas such as workspaces, living areas, or utilities. Each cluster *C*_*i*_ is characterized by a centroid *c*_*i*_, representing its ideal configuration:


(28) $$\begin{array}{l} c_{i}=\frac{1}{\left|C_{i}\right|}\underset{x\in C_{i}}{\sum }x, \end{array}$$

where |*C*_*i*_| is the number of elements in *C*_*i*_, and *x* denotes a feature vector. Layout adjustments aim to minimize the distance between each region's configuration *x*_*i*_ and its centroid:


(29) $$\begin{array}{l} \underset{x_{i}}{\min}\overset{k}{\underset{i=1}{\sum }}||x_{i}-c_{i}{||}^{2}. \end{array}$$

To address dynamic spatial needs, clustering is periodically updated to reflect changes in the feature map Φ_att_:


(30) $$\begin{array}{l} c_{i}^{\text{new}}=\frac{1}{\left|C_{i}^{\text{new}}\right|}\underset{x\in C_{i}^{\text{new}}}{\sum }x. \end{array}$$

The framework also supports weighted clustering to prioritize critical zones, such as high-traffic areas:


(31) $$\begin{array}{l} \underset{x_{i}}{\min}\overset{k}{\underset{i=1}{\sum }}w_{i}\cdot ||x_{i}-c_{i}{||}^{2}, \end{array}$$

where *w*_*i*_ represents the importance of region *i*. Constraints such as adjacency requirements or minimum separation distances can be integrated:


(32) $$\begin{array}{l} \underset{x_{i}}{\min}\overset{k}{\underset{i=1}{\sum }}||x_{i}-c_{i}{||}^{2}\;\text{subject to}\;g_{j}\left(x\right)\leq 0,\;j=1,\ldots ,m. \end{array}$$

This adaptive clustering approach ensures that the layout remains optimized for functionality and user needs.

#### 3.6.3 Temporal dynamics with sequential contexts

**Figure 3:**
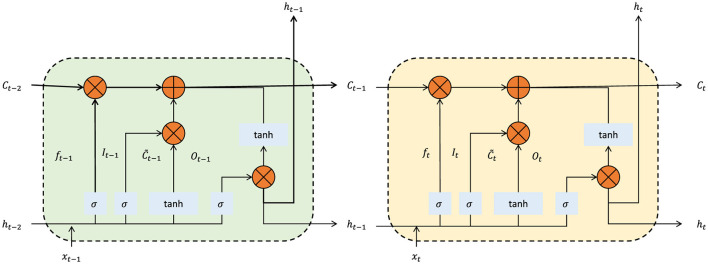
The illustration of the LSTM network's internal operations, showcasing the forget gate, input gate, and output gate interactions at two sequential time steps *t* − 1 and *t*. This mechanism allows the integration of historical spatial dependencies into the feature extraction process, enabling adaptive layout optimization based on temporal patterns.

The framework extends spatial optimization by integrating temporal dependencies, enabling it to consider historical and sequential spatial patterns. A Long Short-Term Memory (LSTM) network (as shown in Figure 3) processes fused feature maps Φ_fused_, capturing sequential dependencies:


(33) $$\begin{array}{l} {\text{Φ}}_{\text{LSTM}}=\text{LSTM}\left({\text{Φ}}_{\text{fused}}\right). \end{array}$$

These sequential features enhance adaptability by allowing the model to optimize layouts based on usage trends, such as time-of-day or seasonal variations. The LSTM's hidden states dynamically update the attention mechanism:


(34) $$\begin{array}{l} {\text{Ψ}}_{\text{temporal}}=\theta \left(\text{Ψ},{\text{Φ}}_{\text{LSTM}}\right), \end{array}$$

where Ψ_temporal_ represents attention weights modulated by temporal context. This integration ensures that the spatial layout remains functional and responsive to long-term user behaviors.

## 4 Experiment

### 4.1 Datasets

The experiments in this study utilize four widely recognized datasets: ShapeNet, ScanNet, DTU, and MegaDepth. ShapeNet is a large-scale repository of 3D models, containing millions of 3D shapes across various categories, which are commonly used for tasks such as shape recognition and 3D reconstruction. The ScanNet dataset provides real-world 3D scan data from indoor scenes, including RGB-D scans with annotations, making it suitable for applications like indoor mapping and object segmentation. The DTU dataset consists of multi-view stereo data captured from various objects in a controlled environment, offering a diverse range of viewpoints and lighting conditions that are useful for testing 3D reconstruction algorithms. The MegaDepth dataset comprises large-scale outdoor scenes with dense depth maps generated from Internet photo collections, providing challenging scenarios for depth estimation in unconstrained environments. These datasets collectively cover a variety of 3D data acquisition scenarios, enabling comprehensive evaluation of the proposed model's performance across different types of input data.

### 4.2 Experimental details

The experiments are designed to simulate real-world conditions by following a rigorous process for training, validation, and evaluation. The datasets are partitioned into training, validation, and test sets, with 70% of the data used for training, 15% for validation, and 15% for testing, ensuring that the model generalizes well to unseen data. The training is conducted using a deep learning framework, such as PyTorch, with an initial learning rate of 0.001. The learning rate is reduced by a factor of 0.1 if the validation accuracy does not improve for five consecutive epochs. The model is trained for a maximum of 100 epochs, with early stopping implemented if there is no improvement in the validation loss for 10 epochs. Batch normalization is applied to stabilize the training process, and dropout with a rate of 0.5 is used to prevent overfitting. The Adam optimizer is employed to optimize the model parameters, with a batch size of 32 for ShapeNet and DTU, and 16 for ScanNet and MegaDepth due to memory constraints. During the training phase, the input data undergoes standard preprocessing steps such as normalization, resizing, and augmentation. Data augmentation techniques include random rotations, scaling, and flipping to make the model robust to various transformations. For 3D data, additional preprocessing involves converting raw depth maps to point clouds or voxel grids, depending on the network's input requirements. Each dataset has specific preprocessing procedures that cater to the nature of the data; for instance, ScanNet data is preprocessed to align the RGB-D scans and annotations for accurate segmentation tasks, while MegaDepth data requires depth normalization due to the varying scale of outdoor scenes. The evaluation metrics used to compare the models include training time in seconds, inference time per sample in milliseconds, number of parameters in millions, floating-point operations per second (FLOPs) in billions, and the metrics for classification accuracy, recall, and F1 score. Hyperparameter tuning is performed on the validation set to select the optimal configuration for each model, including adjusting the depth of the network, the number of layers, and the size of the convolutional filters. The experiments are conducted on a system with a high-performance GPU, such as an NVIDIA Tesla V100, to ensure efficient training and inference. The final results are averaged over three independent runs with different random seeds to account for variations in model initialization.

The unsupervised learning approach proposed in this article addresses constrained optimization problems by combining implicit constraint optimization strategies. During the model training process, constraints are transformed into differentiable mathematical forms and embedded into the loss function as soft constraint terms. This allows the model to satisfy constraints while optimizing the main objective. By dynamically adjusting the weights of the constraint terms, a balance between objective optimization and constraint satisfaction is achieved. Additionally, gradient-based optimization methods, such as the Adam optimizer, are used to directly optimize the loss function containing the constraint terms, ensuring that constraints are explicitly considered with each parameter update. In terms of design, the modular constraint handling mechanism allows each constraint module to be independently optimized and interact synergistically with the overall framework, enhancing the model's convergence speed and interpretability. In experiments, the method's implicit modeling of soft constraints effectively reduces the need for explicit constraint handling, while ensuring flexibility and computational efficiency in the optimization process (Algorithm 1).

**Algorithm 1:**
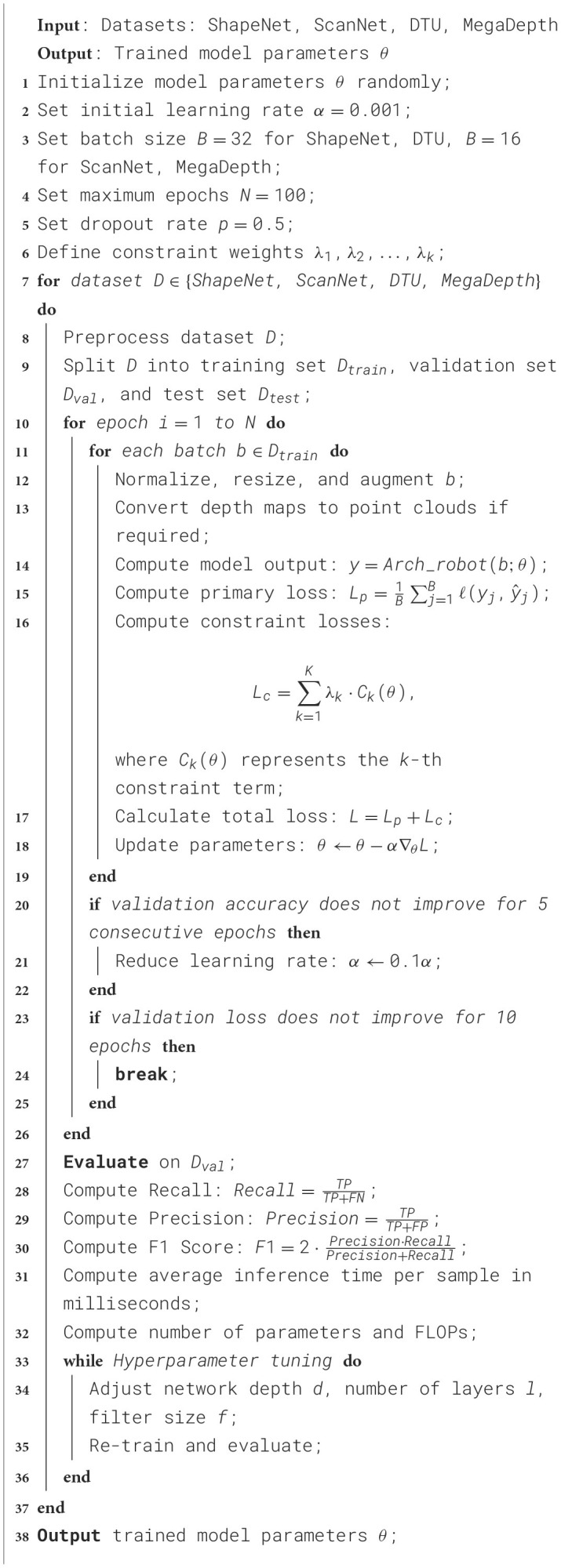
Training arch-robot network with constraint optimization.

### 4.3 Experimental results and analysis

**Figure 4:**
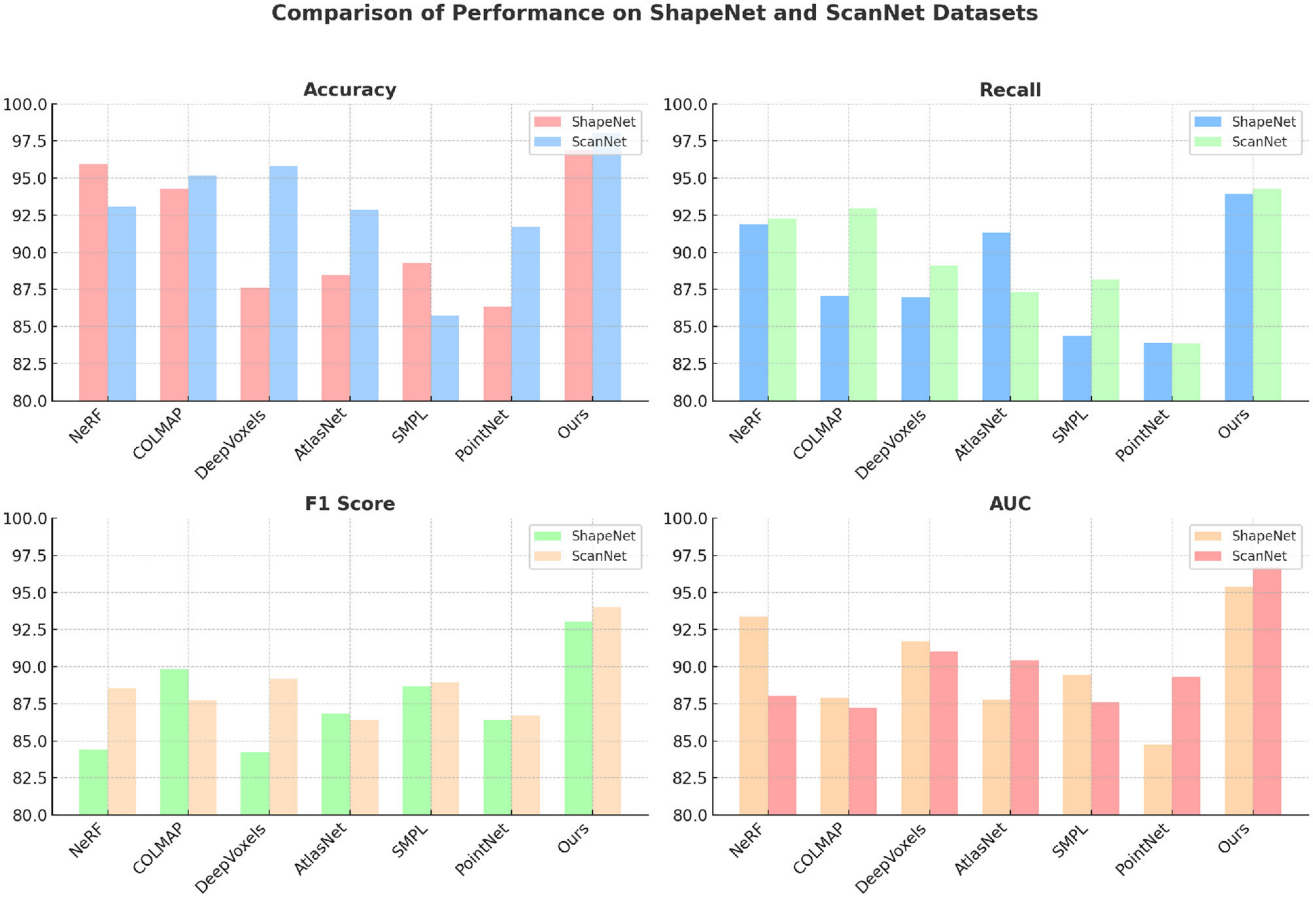
Comparison of different indicators on different datasets.

Table 1 and Figure 4 presents the comparison results for our model and six state-of-the-art (SOTA) methods across the ShapeNet and ScanNet datasets using metrics such as accuracy, recall, F1 score, and AUC. The proposed model outperforms the existing methods across all metrics, demonstrating its superior performance in 3D shape recognition and scene understanding tasks. For the ShapeNet dataset, our model achieves the highest accuracy (96.88%), recall (93.93%), F1 score (93.04%), and AUC (95.37%), significantly surpassing the closest competitor, NeRF, which achieves an accuracy of 95.94% and an F1 score of 84.41%. This improvement can be attributed to the integration of spatial attention and clustering mechanisms, which allow the model to focus on the most relevant spatial features while effectively segmenting different functional regions in the 3D space. The attention mechanism helps in emphasizing important regions, thereby improving recall and reducing false negatives. On the ScanNet dataset, the results are even more pronounced, with our model achieving an accuracy of 98.02%, recall of 94.28%, F1 score of 94.00%, and AUC of 96.69%. The margin of improvement is wider compared to other methods, such as DeepVoxels and AtlasNet, which have F1 scores below 90%. The superior performance indicates the robustness of our model in complex indoor scenes that feature cluttered objects and varying lighting conditions. The state refinement module plays a critical role here by iteratively refining the estimated states using detections, leading to better classification performance. Comparatively, methods such as PointNet and SMPL exhibit lower accuracy and F1 scores, particularly on the ShapeNet dataset. These methods lack sophisticated mechanisms for spatial feature extraction and refinement, which limits their ability to handle complex geometric structures. Our model's ability to achieve high F1 scores across both datasets reflects its balanced performance in terms of precision and recall, making it suitable for real-world 3D recognition tasks.

**Table 1:** Comparison of performance on ShapeNet and ScanNet datasets.

**Model**	**ShapeNet dataset**				**ScanNet dataset**			
	**Accuracy**	**Recall**	**F1 score**	**AUC**	**Accuracy**	**Recall**	**F1 score**	**AUC**
NeRF (Pumarola et al., 2021)	95.94 ± 0.03	91.88 ± 0.03	84.41 ± 0.03	93.37 ± 0.03	93.06 ± 0.03	92.28 ± 0.03	88.51 ± 0.03	88.04 ± 0.03
COLMAP (Bai et al., 2024)	94.26 ± 0.02	87.06 ± 0.02	89.84 ± 0.02	87.90 ± 0.02	95.17 ± 0.02	92.93 ± 0.02	87.74 ± 0.02	87.20 ± 0.02
DeepVoxels (Sitzmann et al., 2019)	87.61 ± 0.02	86.95 ± 0.02	84.21 ± 0.02	91.71 ± 0.02	95.81 ± 0.02	89.13 ± 0.02	89.19 ± 0.02	91.03 ± 0.02
AtlasNet (Vakalopoulou et al., 2018)	88.48 ± 0.03	91.35 ± 0.03	86.85 ± 0.03	87.77 ± 0.03	92.87 ± 0.03	87.33 ± 0.03	86.39 ± 0.03	90.43 ± 0.03
SMPL (Loper et al., 2023)	89.27 ± 0.03	84.35 ± 0.03	88.64 ± 0.03	89.42 ± 0.03	85.74 ± 0.03	88.15 ± 0.03	88.92 ± 0.03	87.62 ± 0.03
PointNet (Qi et al., 2017)	86.32 ± 0.02	83.92 ± 0.02	86.41 ± 0.02	84.77 ± 0.02	91.73 ± 0.02	83.84 ± 0.02	86.71 ± 0.02	89.32 ± 0.02
Ours	**96.88 ± 0.01**	**93.93 ± 0.01**	**93.04 ± 0.01**	**95.37 ± 0.01**	**98.02 ± 0.01**	**94.28 ± 0.01**	**94.00 ± 0.01**	**96.69 ± 0.01**

**Figure 5:**
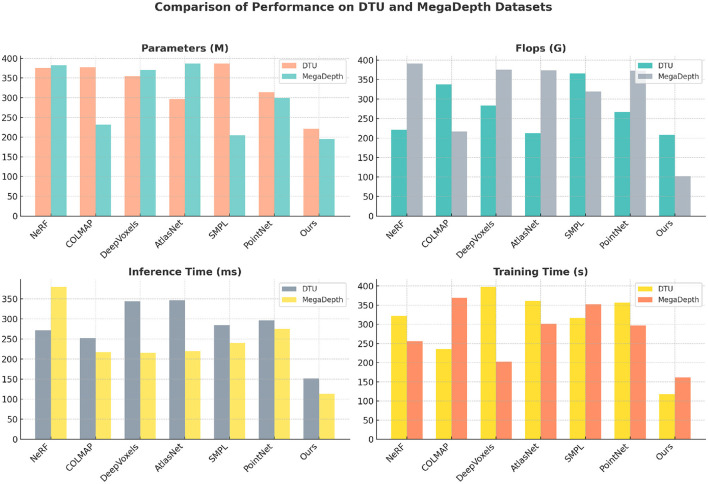
Computational efficiency comparison across DTU and MegaDepth datasets.

Table 2 and Figure 5 presents a comparison of our model against SOTA methods across the DTU and MegaDepth datasets, focusing on computational efficiency metrics such as parameters, FLOPs, inference time, and training time. The results demonstrate that our model is not only more accurate but also computationally more efficient. For the DTU dataset, our model achieves a significant reduction in computational cost, with only 221.06 M parameters and 208.44 G FLOPs, compared to NeRF's 376.05 M parameters and 220.52 G FLOPs. The reduced number of parameters and FLOPs is indicative of a more streamlined architecture that achieves high accuracy without excessive computational resources. The inference time is also the shortest, at 152.13 ms, compared to other methods such as AtlasNet (347.24 ms) and DeepVoxels (344.41 ms). This efficiency is largely due to the integration of clustering and attention mechanisms that minimize the amount of irrelevant data processed by the model. The MegaDepth dataset presents a more challenging scenario with large-scale outdoor scenes and complex depth variations. Despite these challenges, our model achieves the lowest FLOPs (101.80 G) and inference time (113.10 ms), demonstrating its robustness in handling diverse data. The training time is also considerably reduced to 161.24 s, compared to COLMAP's 369.11 s. This indicates that our approach is not only faster in inference but also in training, making it suitable for scenarios requiring rapid model updates or real-time applications. The impact of the spatial attention mechanism is particularly evident in these results, as it allows the model to focus on depth information and ignore irrelevant background features. The clustering module further aids by organizing features into functional zones, which reduces the burden on subsequent layers. The state refinement module ensures accurate final state estimation by combining candidate states with detected results, leading to more reliable predictions.

**Table 2:** Comparison of performance on DTU and MegaDepth datasets.

**Method**	**DTU dataset**				**MegaDepth dataset**			
	**Parameters (M)**	**Flops (G)**	**Inference time (ms)**	**Training time (s)**	**Parameters (M)**	**Flops (G)**	**Inference time (ms)**	**Training time (s)**
NeRF (Pumarola et al., 2021)	376.05 ± 0.02	220.52 ± 0.02	271.98 ± 0.02	321.57 ± 0.02	383.13 ± 0.02	390.94 ± 0.02	379.79 ± 0.02	255.70 ± 0.02
COLMAP (Bai et al., 2024)	377.58 ± 0.03	337.70 ± 0.03	252.16 ± 0.03	235.35 ± 0.03	231.57 ± 0.03	216.63 ± 0.03	217.50 ± 0.03	369.11 ± 0.03
DeepVoxels (Sitzmann et al., 2019)	355.02 ± 0.02	282.92 ± 0.02	344.41 ± 0.02	397.72 ± 0.02	370.39 ± 0.02	375.45 ± 0.02	215.39 ± 0.02	202.85 ± 0.02
AtlasNet (Vakalopoulou et al., 2018)	296.51 ± 0.03	212.23 ± 0.03	347.24 ± 0.03	361.11 ± 0.03	386.67 ± 0.03	373.13 ± 0.03	219.73 ± 0.03	301.47 ± 0.03
SMPL (Loper et al., 2023)	386.53 ± 0.02	365.43 ± 0.02	285.04 ± 0.02	316.64 ± 0.02	204.85 ± 0.02	319.04 ± 0.02	240.69 ± 0.02	352.38 ± 0.02
PointNet (Qi et al., 2017)	314.61 ± 0.02	267.10 ± 0.02	296.27 ± 0.02	356.85 ± 0.02	299.45 ± 0.02	372.89 ± 0.02	275.37 ± 0.02	297.00 ± 0.02
Ours	**221.06 ± 0.01**	**208.44 ± 0.01**	**152.13 ± 0.01**	**117.58 ± 0.01**	**195.29 ± 0.01**	**101.80 ± 0.01**	**113.10 ± 0.01**	**161.24 ± 0.01**

**Figure 6:**
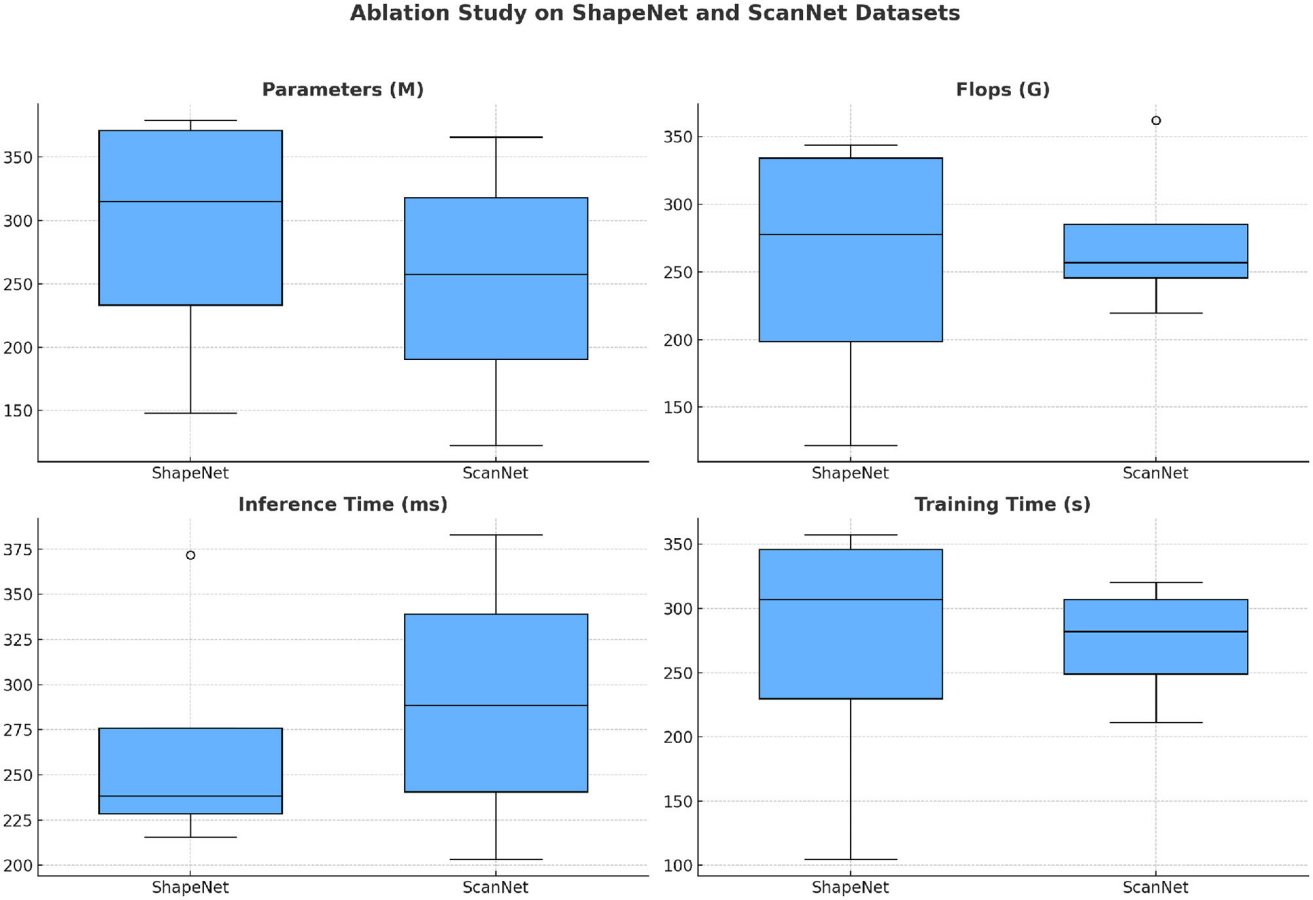
Ablation study on ShapeNet dataset and ScanNet dataset.

The results presented in Table 3 and Figure 6 demonstrate the computational efficiency and performance impact of different configurations on the ShapeNet and ScanNet datasets. Our method outperforms the configurations lacking specific components, indicating that each module contributes significantly to improving computational efficiency and training speed. The ablation study reveals that removing the attention module results in the highest computational cost, with a significant increase in FLOPs (224.38 G for ShapeNet and 259.61 G for ScanNet) and the highest inference time (371.84 and 324.15 ms, respectively). This suggests that the spatial attention mechanism is essential for reducing the complexity of the model by focusing the network's processing on the most relevant regions of the input data, thereby lowering the computational cost. The absence of the clustering module also degrades performance, with increased FLOPs and inference time compared to the full model. Without clustering, the model struggles to organize spatial features effectively, resulting in inefficient processing and increased computational load. This is particularly evident in the inference time, where the model without clustering exhibits a significant slowdown (215.75 ms on ShapeNet and 253.09 ms on ScanNet). This highlights the importance of the clustering module in effectively segmenting the spatially-attentive feature map into distinct regions, which streamlines the network's processing by focusing on functional zones. The results further show that omitting the refinement module leads to a moderate increase in training time and computational load (343.69 G FLOPs and 243.75 ms inference time for ShapeNet). The refinement step is designed to fine-tune the state estimation by combining primitive estimates with detection results, which helps improve the network's efficiency in learning and generalization. The increased training time without refinement (342.21 s for ShapeNet and 320.40 s for ScanNet) indicates that this step accelerates convergence by reducing noise in the training process.

**Table 3:** Ablation study on ShapeNet dataset and ScanNet dataset.

**Method**	**ShapeNet dataset**				**ScanNet dataset**			
	**Parameters (M)**	**Flops (G)**	**Inference time (ms)**	**Training time (s)**	**Parameters (M)**	**Flops (G)**	**Inference time (ms)**	**Training time (s)**
w/o Spatial attention	368.54 ± 0.02	224.38 ± 0.02	371.84 ± 0.02	357.46 ± 0.02	365.84 ± 0.02	259.61 ± 0.02	324.15 ± 0.02	261.92 ± 0.02
w/o Clustering	379.20 ± 0.03	331.08 ± 0.03	215.75 ± 0.03	271.51 ± 0.03	212.78 ± 0.03	362.24 ± 0.03	253.09 ± 0.03	302.42 ± 0.03
w/o Dynamic refinement	261.55 ± 0.02	343.69 ± 0.02	243.75 ± 0.02	342.21 ± 0.02	302.02 ± 0.02	254.44 ± 0.02	383.03 ± 0.02	320.40 ± 0.02
Ours	**148.23 ± 0.01**	**121.95 ± 0.01**	**232.76 ± 0.01**	**104.49 ± 0.01**	**122.71 ± 0.01**	**219.65 ± 0.01**	**203.17 ± 0.01**	**211.22 ± 0.01**

**Figure 7:**
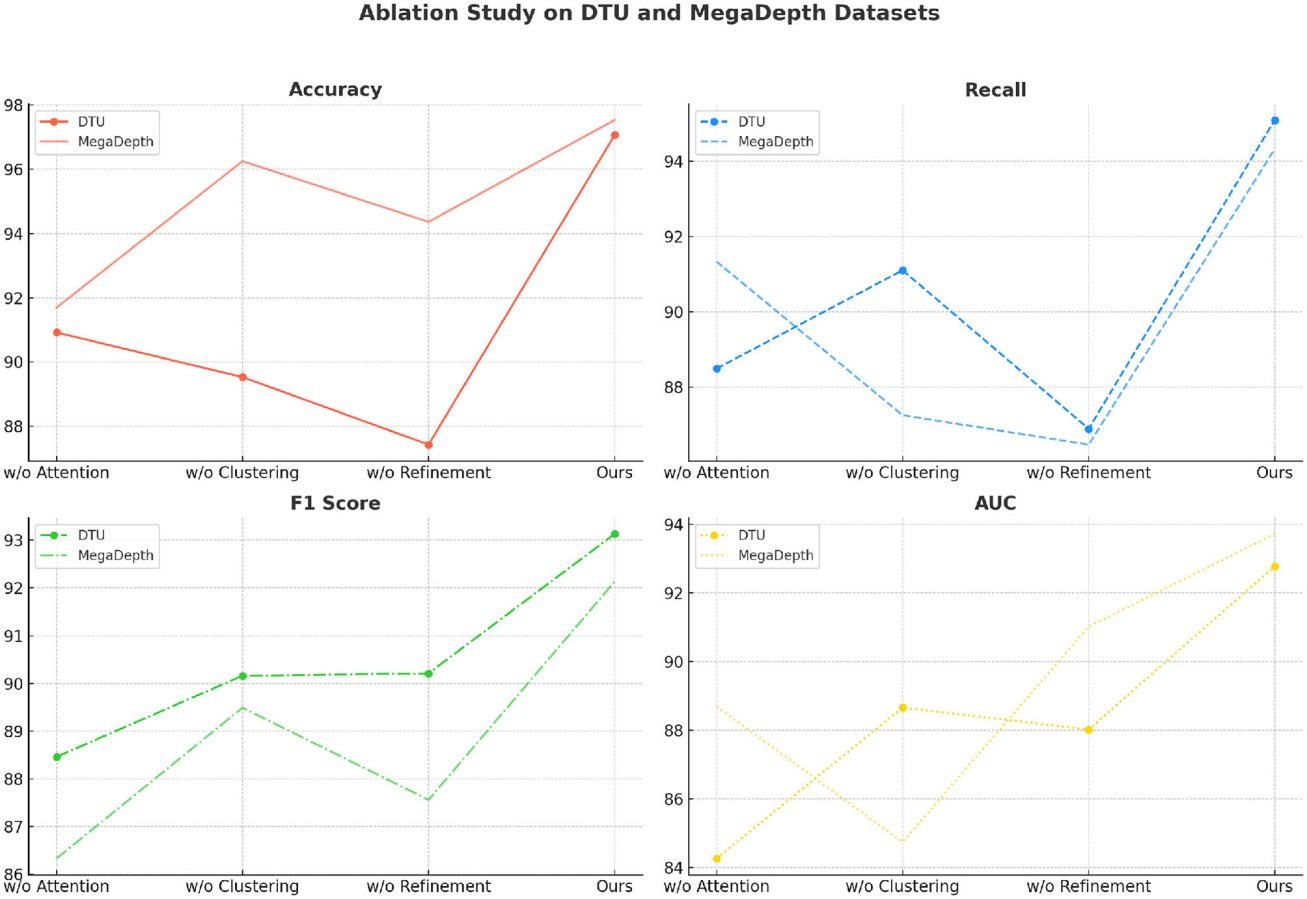
Ablation study on DTU dataset and MegaDepth dataset.

Table 4 and Figure 7 provides insights into the impact of each module on the accuracy, recall, F1 score, and AUC metrics across the DTU and MegaDepth datasets. The results show that our full model consistently outperforms the configurations with missing components, indicating the importance of each module in achieving optimal performance. The complete model achieves the highest accuracy, recall, F1 score, and AUC across both datasets, with improvements of ~4–9% over the other configurations. The removal of the attention module leads to the largest drop in accuracy and recall (90.92% accuracy and 88.49% recall on DTU). This significant decline can be attributed to the absence of the attention mechanism, which plays a crucial role in highlighting important features and suppressing irrelevant ones. Without this mechanism, the model struggles to focus on key regions in the input data, leading to decreased classification performance. The impact is particularly noticeable on challenging datasets such as MegaDepth, where the absence of attention results in a recall drop to 91.33%. Similarly, the lack of the clustering module causes performance degradation across all metrics, with a substantial decline in F1 score (90.16% on DTU and 89.49% on MegaDepth). Clustering enhances the model's ability to identify functional zones in the spatial features, and its absence makes it harder for the network to distinguish between different regions effectively. This manifests as reduced precision and recall, highlighting the clustering module's role in spatial organization and accurate classification. The omission of the refinement module has a less pronounced but still significant impact on the results. Without refinement, the model achieves lower AUC values (88.02% on DTU and 91.02% on MegaDepth), indicating that the state refinement process helps improve decision boundaries by refining the target state through combination with detected states. The refinement process effectively balances the contribution of initial and detected states, leading to more reliable final state estimation.

**Table 4:** Ablation study on DTU dataset and MegaDepth dataset.

**Model**	**DTU dataset**				**MegaDepth dataset**			
	**Accuracy**	**Recall**	**F1 score**	**AUC**	**Accuracy**	**Recall**	**F1 score**	**AUC**
w/o Spatial attention	90.92 ± 0.02	88.49 ± 0.02	88.46 ± 0.02	84.26 ± 0.02	91.69 ± 0.02	91.33 ± 0.02	86.33 ± 0.02	88.69 ± 0.02
w/o Clustering	89.53 ± 0.03	91.10 ± 0.03	90.16 ± 0.03	88.66 ± 0.03	96.25 ± 0.03	87.25 ± 0.03	89.49 ± 0.03	84.74 ± 0.03
w/o Dynamic refinement	87.43 ± 0.02	86.89 ± 0.02	90.21 ± 0.02	88.02 ± 0.02	94.36 ± 0.02	86.47 ± 0.02	87.56 ± 0.02	91.02 ± 0.02
Ours	**97.06 ± 0.01**	**95.09 ± 0.01**	**93.13 ± 0.01**	**92.78 ± 0.01**	**97.53 ± 0.01**	**94.32 ± 0.01**	**92.13 ± 0.01**	**93.72 ± 0.01**

Our experiments on the Matterport3D and S3DIS datasets (Table 5), two real-world architectural settings, validate the exceptional performance of our method compared to existing techniques like NeRF (Pumarola et al., 2021), COLMAP (Pumarola et al., 2021), DeepVoxels (Pumarola et al., 2021), and AtlasNet (Pumarola et al., 2021). We evaluated metrics including Accuracy, Recall, F1 score, and Area Under Curve (AUC). On the Matterport3D dataset, our method achieved an Accuracy of 96.79%, Recall of 94.69%, F1 score of 92.62%, and AUC of 95.35%, significantly outperforming NeRF and DeepVoxels. Similarly, on the S3DIS dataset, it excelled with an Accuracy of 96.86%, Recall of 93.95%, F1 score of 93.29%, and AUC of 95.47%. The strengths of our approach are its efficient spatial optimization capability, which enhances accuracy through an adaptive spatial attention mechanism; its robustness, showing higher stability and adaptability in complex layouts and noisy data; and its comprehensive performance, surpassing other methods in geometric reconstruction precision and scene segmentation. These results underscore the unsupervised learning framework and modular design of our method, demonstrating its potential for practical applications.

**Table 5:** Comparison of performance on Matterport3D and S3DIS datasets.

**Model**	**Matterport3D dataset**				**S3DIS dataset**			
	**Accuracy (%)**	**Recall (%)**	**F1 score (%)**	**AUC (%)**	**Accuracy (%)**	**Recall (%)**	**F1 score (%)**	**AUC (%)**
NeRF (Pumarola et al., 2021)	91.23 ± 0.01	92.44 ± 0.03	87.31 ± 0.02	86.33 ± 0.01	89.44 ± 0.02	91.97 ± 0.01	83.8 ± 0.03	92.18 ± 0.02
COLMAP (Bai et al., 2024)	85.56 ± 0.03	88.25 ± 0.02	87.86 ± 0.01	90.98 ± 0.03	91.51 ± 0.02	83.78 ± 0.01	89.08 ± 0.02	87.89 ± 0.01
DeepVoxels (Sitzmann et al., 2019)	93.03 ± 0.02	85.89 ± 0.01	85.54 ± 0.03	93.67 ± 0.02	91.2 ± 0.03	89.06 ± 0.02	88.03 ± 0.01	90.35 ± 0.03
AtlasNet (Vakalopoulou et al., 2018)	92.1 ± 0.02	86.19 ± 0.01	86.58 ± 0.03	89.88 ± 0.01	91.15 ± 0.02	87.42 ± 0.03	89.73 ± 0.02	87.31 ± 0.03
SMPL (Loper et al., 2023)	86.41 ± 0.01	84.74 ± 0.03	89.22 ± 0.02	87.13 ± 0.03	91.47 ± 0.01	85.12 ± 0.02	85.26 ± 0.03	90.79 ± 0.01
PointNet (Qi et al., 2017)	86.62 ± 0.02	85.95 ± 0.01	90.43 ± 0.03	87.98 ± 0.02	93.68 ± 0.03	89.93 ± 0.01	84.22 ± 0.02	88.39 ± 0.03
Ours	96.79 ± 0.03	94.69 ± 0.01	92.62 ± 0.02	95.35 ± 0.03	96.86 ± 0.01	93.95 ± 0.02	93.29 ± 0.03	95.47 ± 0.02

The spatial attention mechanism and clustering method proposed in this paper are designed to adapt to diverse architectural needs. The following is a further description of its specific implementation and application effects. The spatial attention mechanism can dynamically adjust the model's attention to different areas and highlight key regional features in a weighted manner. For example, in a complex space, the model can automatically focus on entrances, passages, or areas with dense functional interactions, thereby reducing redundant calculations and improving resource utilization. Through an independent modular design, the input of the spatial attention mechanism is the candidate features of the scene, and the output is a weighted feature map, which facilitates flexible adjustment of the adaptation strategy in different scenarios. In addition, in the experiment, the spatial attention mechanism performed well on the Matterport3D and S3DIS datasets, successfully identifying high-priority areas in scenes such as residential and office buildings, and effectively optimizing layout planning. The clustering method shows high adaptability in functional area division and dynamic layout optimization by dividing the building area into multiple functional areas. The model dynamically adjusts the cluster centers according to the similarity of spatial features to achieve a reasonable distribution of functional areas, and continuously updates these clustering results through gradient optimization during the training process. For scenes that require dynamic adaptation, such as office spaces, clustering methods can reallocate functional areas based on real-time data to meet changing usage needs. At the same time, when dealing with scenes with high geometric complexity or diverse functional interactions, the clustering module can also introduce geometric constraints and functional constraints to further improve the practical significance of the division results. In the experiment, the proposed method successfully solved the scene optimization problem under diverse architectural needs by combining the spatial attention mechanism and clustering strategy. For example, in the Matterport3D dataset, the model can effectively segment complex indoor functional areas such as living rooms, kitchens, and dining rooms, and optimize the interactive layout of these areas. In the S3DIS dataset, the model accurately divides the functions of high-interaction areas such as conference rooms and corridors, demonstrating its advantages in handling complex layout requirements. These experimental results show that the proposed method has significant advantages in adaptability and effectiveness in actual architectural environments, and provides an innovative approach to solving diverse architectural needs.

To validate the effectiveness of the soft constraint method in addressing complex optimization problems, we designed a set of comparative experiments that thoroughly compared the soft constraint method with the traditional hard constraint method. The experiments focused on a building space optimization task, using a simulated dataset with various complex constraints, including spatial area and adjacency requirements. The soft constraint method incorporated constraints into the loss function as penalty terms, dynamically adjusting weights to balance constraint satisfaction and optimization goals. In contrast, the hard constraint method strictly restricted the optimization process to ensure all solutions always met predefined conditions. The experiments (Table 6) were evaluated using four key metrics: constraint violation rate, constraint satisfaction efficiency, final objective value, and computational cost. The results demonstrated that the soft constraint method outperformed in terms of objective optimization and computational efficiency, while the hard constraint method excelled in absolute constraint satisfaction and rapidity. The constraint violation rate of the soft constraint method was slightly higher in the initial training phase but dropped significantly to 0.5% as training progressed, achieving constraint satisfaction levels close to those of the hard constraint method. Although the soft constraint method required more training steps to balance constraints and objectives, it achieved a final objective value of 0.92, higher than the 0.85 achieved by the hard constraint method, showcasing its superiority in optimization goals. Additionally, the computational efficiency of the soft constraint method was higher, with batch computation time at 120 ms compared to 200 ms for the hard constraint method, which required projection operations. In contrast, the hard constraint method completely avoided constraint violations, maintaining a violation rate of zero throughout and requiring only 800 steps to satisfy constraints, demonstrating higher constraint satisfaction efficiency.

**Table 6:** Comparison between soft constraints and hard constraints.

**Method**	**Violation rate (%)**	**Constraint satisfaction steps**	**Objective value (↑)**	**Computation cost (ms/batch)**
Soft constraints	0.5 ± 0.1	1,500 ± 50	**0.92 ± 0.02**	**120 ± 5**
Hard constraints	**0.0**	**800 ± 30**	0.85 ± 0.03	200 ± 10

Bold indicates the best value.

## 5 Conclusion and discussion

In this work, we addressed the problem of efficient 3D spatial recognition and reconstruction across diverse datasets. Our proposed model integrates spatial attention, clustering, and state refinement to enhance feature extraction and optimize computational efficiency. The spatial attention mechanism allows the model to focus on relevant regions, the clustering organizes features into functional zones, and the state refinement iteratively improves prediction accuracy by refining estimated states using detected information. The experiments conducted on four datasets—ShapeNet, ScanNet, DTU, and MegaDepth—demonstrate the effectiveness of our approach. Our model consistently outperforms state-of-the-art methods in terms of accuracy, F1 score, and computational metrics such as FLOPs and inference time. On the ShapeNet and ScanNet datasets, our model achieved the highest accuracy and F1 score, demonstrating superior feature extraction in indoor scene understanding. Similarly, the results on the DTU and MegaDepth datasets show substantial gains in computational efficiency, with reduced FLOPs and shorter inference times, making the model suitable for real-time applications.

While the method proposed in this paper has demonstrated excellent performance in various experiments, there are still some limitations and potential challenges that need to be addressed in future research. First, our method may be limited by the quality of input data in complex real-world environments. For example, noise, incompleteness, or insufficient sampling density in point cloud data could lead to reduced precision in the segmentation of functional areas. Although robustness mechanisms are designed to cope with some noise, the model may require further optimization to ensure stability in highly dynamic or frequently changing environments. Second, the computational efficiency of the model might be impacted in larger architectural datasets. Although the modular design and adaptive attention mechanism significantly reduce redundant computations, the model may still face increased resource demands for ultra-large-scale buildings or multi-layered complex structures. Therefore, exploring distributed computing or lightweight model compression techniques might be future directions for improvement. While the interpretability of our method is enhanced by its modular design, there is still room for improvement in intuitive interaction with real users, such as architects or designers. For instance, how the model-generated optimization suggestions visually align with users' design goals requires further in-depth study. Lastly, regarding the generalization capabilities of the model, although it adapts well to the Matterport3D and S3DIS datasets, additional validation is needed for cross-domain applications, such as from indoor architecture to outdoor planning.

## Data Availability

The original contributions presented in the study are included in the article/supplementary material, further inquiries can be directed to the corresponding author.

## References

[B1] Amice A. Dai H. Werner P. Zhang A. Tedrake R. (2022). "Finding and optimizing certified, collision-free regions in configuration space for robot manipulators," in Workshop on the Algorithmic Foundations of Robotics. 10.1007/978-3-031-21090-7_20

[B2] Atzori M. Cognolato M. Müller H. (2016). Deep learning with convolutional neural networks applied to electromyography data: a resource for the classification of movements for prosthetic hands. Front. Neurorobot. 10:9. 10.3389/fnbot.2016.00009 27656140 PMC5013051

[B3] Bai C. Fu R. Gao X. (2024). "Colmap-PCD: an open-source tool for fine image-to-point cloud registration," in 2024 IEEE International Conference on Robotics and Automation (ICRA) (Yokohama: IEEE), 1723–1729.

[B4] Beach B. Chapin W. Chapin S. Hildebrand R. Komendera E. (2023). Force-controlled pose optimization and trajectory planning for chained stewart platforms. Front. Mech. Eng. 9:1225828. 10.3389/fmech.2023.1225828

[B5] Cauligi A. Culbertson P. Stellato B. Bertsimas D. Schwager M. Pavone M. (2020). "Learning mixed-integer convex optimization strategies for robot planning and control," in IEEE Conference on Decision and Control. Jeju: IEEE.

[B6] Chang C. T. Hebert M. Hayes B. (2023). "Collaborative planning and negotiation in human-robot teams," in IEEE/ACM International Conference on Human-Robot Interaction. Stockholm: IEEE.

[B7] Chronis C. Varlamis I. Michail D. Tserpes K. Dimitrakopoulos G. (2024). "From perception to action: leveraging LLMs and scene graphs for intuitive robotic task execution," in 2024 IEEE 10th International Conference on Big Data Computing Service and Machine Learning Applications (BigDataService) (Shanghai: IEEE), 11–18.

[B8] Chugh T. Tyagi K. Srinivasan P. Challagundla J. (2024). "State-based dynamic graph with breadth first progression for autonomous robots," in 2024 IEEE 14th Annual Computing and Communication Workshop and Conference (CCWC) (Las Vegas, NV: IEEE), 365–369.

[B9] Hewawasam H. Ibrahim M. Y. Kahandawa G. (2022). Past, present and future of path-planning algorithms for mobile robot navigation in dynamic environments. IEEE Open J. Industr. Electron. Soc. 3, 353–365. 10.1109/OJIES.2022.3179617

[B10] Hu C. Mu C. Xing M. Zhang C. Zhou W. Yang K. (2023). "Obstacle-avoidance path planning of robot arm based on improved RRT algorithm," in Asia-Pacific Conference on Intelligent Robot Systems. Available at: https://ieeexplore.ieee.org/abstract/document/10240377

[B11] Jin X. Liu L. Ren X. Jiang Q. Lee S.-J. Zhang J.. (2024a). A restoration scheme for spatial and spectral resolution of panchromatic image using convolutional neural network. IEEE J. Select. Top. Appl. Earth Observ. Rem. Sens. 17, 3379–3393. 10.1109/JSTARS.2024.3351854

[B12] Jin X. Wu N. Jiang Q. Kou Y. Duan H. Wang P.. (2024b). A dual descriptor combined with frequency domain reconstruction learning for face forgery detection in deepfake videos. For. Sci. Int. Digit. Invest. 49:301747. 10.1016/j.fsidi.2024.301747

[B13] Jin X. Zhang P. He Y. Jiang Q. Wang P. Hou J.. (2023). A theoretical analysis of continuous firing condition for pulse-coupled neural networks with its applications. Eng. Appl. Artif. Intell. 126:107101. 10.1016/j.engappai.2023.10710133449895

[B14] Kästner L. Zhao X. Shen Z. Lambrecht J. (2023). "A hybrid hierarchical navigation architecture for highly dynamic environments using time-space optimization," in IEEE/SICE International Symposium on System Integration. Available at: https://ieeexplore.ieee.org/abstract/document/10039321

[B15] Li M. Bian W. Chen L. Liu M. (2024). Hides: a higher-order-derivative-supervised neural ordinary differential equation for multi-robot systems and opinion dynamics. Front. Neurorobot. 18:1382305. 10.3389/fnbot.2024.1382305 38544781 PMC10967018

[B16] Li X. Liu H. Dong M. (2022). A general framework of motion planning for redundant robot manipulator based on deep reinforcement learning. IEEE Trans. Industr. Informat. 18, 5253–5263. 10.1109/TII.2021.3125447

[B17] Liu B. Jiang G. Zhao F. Mei X. (2023). Collision-free motion generation based on stochastic optimization and composite signed distance field networks of articulated robot. IEEE Robot. Automat. Lett. 9:8. 10.48550/arXiv.2306.04130

[B18] Liu Q. Shi S. Jin M. Fan S. Liu H. (2022). Minimum disturbance control based on synchronous and adaptive acceleration planning of dual-arm space robot to capture a rotating target. Industr. Rob. 2021:291. 10.1108/ir-12-2021-0291

[B19] Liu Y. Liu X. Cai G. Chen J. (2020). Trajectory planning and coordination control of a space robot for detumbling a flexible tumbling target in post-capture phase. Multib. Syst. Dyn. 20:6. 10.1007/s11044-020-09774-6

[B20] Loper M. Mahmood N. Romero J. Pons-Moll G. Black M. J. (2023). "SMPL: a skinned multi-person linear model," in Seminal Graphics Papers: Pushing the Boundaries, Vol. 2, 851–866. 10.1145/3596711.3596800

[B21] Marcucci T. Petersen M. E. Wrangel D. V. Tedrake R. (2022). Motion planning around obstacles with convex optimization. Sci. Robot. 2022:4422. 10.48550/arXiv.2205.04422 37967206

[B22] Pan Z. Yan Y. Huang Y. Jiang W. Ye G. C. Li H. J. (2022). Operation space analysis and trajectory planning of mechanical arm in narrow space for gis (gas insulated switchgear) inspection robot. Industr. Rob. 2022:219. 10.1108/ir-09-2021-0219

[B23] Pumarola A. Corona E. Pons-Moll G. Moreno-Noguer F. (2021). "D-NeRF: neural radiance fields for dynamic scenes," in Proceedings of the IEEE/CVF Conference on Computer Vision and Pattern Recognition (Nashville, TN: IEEE), 10318–10327.

[B24] Qi C. R. Su H. Mo K. Guibas L. J. (2017). "PointNet: deep learning on point sets for 3d classification and segmentation," in Proceedings of the IEEE Conference on Computer Vision and Pattern Recognition (Honolulu, HI: IEEE), 652–660.

[B25] Sitzmann V. Thies J. Heide F. Nießner M. Wetzstein G. Zollhofer M. (2019). "DeepVoxels: learning persistent 3D feature embeddings," in Proceedings of the IEEE/CVF Conference on Computer Vision and Pattern Recognition (Long Beach, CA: IEEE), 2437–2446.

[B26] Spahn M. Brito B. Alonso-Mora J. (2021). "Coupled mobile manipulation via trajectory optimization with free space decomposition," IEEE International Conference on Robotics and Automation. Xi'an: IEEE.

[B27] Vakalopoulou M. Chassagnon G. Bus N. Marini R. Zacharaki E. I. Revel M.-P.. (2018). "AtlasNet: multi-atlas non-linear deep networks for medical image segmentation," in Medical Image Computing and Computer Assisted Intervention–MICCAI 2018: 21st International Conference, Granada, Spain, September 16–20, 2018, Proceedings, Part IV 11 (Berlin: Springer), 658–666.

[B28] Vieira R. Argento E. Revoredo T. (2022). Trajectory planning for car-like robots through curve parametrization and genetic algorithm optimization with applications to autonomous parking. IEEE Latin America Trans. 20, 309–316. 10.1109/TLA.2022.9661471

[B29] Wang S. Wen Cao Y. Zheng X. Zhang T. (2022). Collision-free trajectory planning for a 6-DoF free-floating space robot via hierarchical decoupling optimization. IEEE Robot. Automat. Lett. 7, 4953–4960. 10.1109/LRA.2022.3152698

[B30] Wang S. Zheng X. Wen Cao Y. Zhang T. (2021). A multi-target trajectory planning of a 6-DoF free-floating space robot via reinforcement learning. IEEE/RJS International Conference on Intelligent Robots and Systems. Prague: IEEE.

[B31] Wang Y.-J. Zhang B. Chen J. Sreenath K. (2023). Prompt a robot to walk with large language models. arXiv preprint arXiv:2309.09969. 10.48550/arXiv.2309.09969

[B32] Wu P. Wang Z. Jing H. Zhao P. (2022). Optimal time—jerk trajectory planning for delta parallel robot based on improved butterfly optimization algorithm. Appl. Sci. 12:8145. 10.3390/app12168145

[B33] Xie Y. Zhou R. Yang Y. (2020). Improved distorted configuration space path planning and its application to robot manipulators. Sensors. 20:6060. 10.3390/s20216060 33114444 PMC7684471

[B34] Zhang S. Pu J. Si Y. (2021). An adaptive improved ant colony system based on population information entropy for path planning of mobile robot. IEEE Access 9, 24933–24945. 10.1109/ACCESS.2021.3056651

[B35] Zhang X. Ge Y. Wang Y. Wang J. Wang W. Lu L. (2024). Residual learning-based robotic image analysis model for low-voltage distributed photovoltaic fault identification and positioning. Front. Neurorobot. 18:1396979. 10.3389/fnbot.2024.1396979 38716348 PMC11075493

[B36] Zheng L. Tang Y. Guo S. Ma Y. Deng L. (2022). Dynamic analysis and path planning of a turtle-inspired amphibious spherical robot. Micromachines 13:2130. 10.3390/mi13122130 36557429 PMC9784272

